# Metabolomic analysis of mouse prefrontal cortex reveals upregulated analytes during wakefulness compared to sleep

**DOI:** 10.1038/s41598-018-29511-6

**Published:** 2018-07-25

**Authors:** Allen K. Bourdon, Giovanna Maria Spano, William Marshall, Michele Bellesi, Giulio Tononi, Pier Andrea Serra, Helen A. Baghdoyan, Ralph Lydic, Shawn R. Campagna, Chiara Cirelli

**Affiliations:** 1 Department of Chemistry, University of Tennessee, Knoxville, TN United States; 2 Department of Psychiatry, University of Wisconsin, Madison, Madison, WI United States; 3 Department of Clinical and Experimental Medicine, University of Sassari, Sassari, Italy; 4 Department of Anesthesiology and Psychology, University of Tennessee, Knoxville, TN United States; 5 Oak Ridge National Laboratory, Oak Ridge, TN United States; 6 Biological and Small Molecule Mass Spectrometry Core, University of Tennessee, Knoxville, TN United States; 7 Department of Experimental and Clinical Medicine, Section of Neuroscience and Cell Biology, Università Politecnica delle Marche, Ancona, Italy

## Abstract

By identifying endogenous molecules in brain extracellular fluid metabolomics can provide insight into the regulatory mechanisms and functions of sleep. Here we studied how the cortical metabolome changes during sleep, sleep deprivation and spontaneous wakefulness. Mice were implanted with electrodes for chronic sleep/wake recording and with microdialysis probes targeting prefrontal and primary motor cortex. Metabolites were measured using ultra performance liquid chromatography-high resolution mass spectrometry. Sleep/wake changes in metabolites were evaluated using partial least squares discriminant analysis, linear mixed effects model analysis of variance, and machine-learning algorithms. More than 30 known metabolites were reliably detected in most samples. When used by a logistic regression classifier, the profile of these metabolites across sleep, spontaneous wake, and enforced wake was sufficient to assign mice to their correct experimental group (pair-wise) in 80–100% of cases. Eleven of these metabolites showed significantly higher levels in awake than in sleeping mice. Some changes extend previous findings (glutamate, homovanillic acid, lactate, pyruvate, tryptophan, uridine), while others are novel (D-gluconate, N-acetyl-beta-alanine, N-acetylglutamine, orotate, succinate/methylmalonate). The upregulation of the *de novo* pyrimidine pathway, gluconate shunt and aerobic glycolysis may reflect a wake-dependent need to promote the synthesis of many essential components, from nucleic acids to synaptic membranes.

## Introduction

Genome-wide transcriptomic studies have identified hundreds of mRNAs that show expression changes in neurons and glia during sleep and wakefulness, independent of circadian time^1–6^. These studies have suggested new hypotheses about the restorative functions of sleep, from synaptic homeostasis to the synthesis of cellular components and membranes^3,4,7^. Proteomic approaches also have been used to investigate functions of sleep, although applying proteomics to brain homogenates is difficult (e.g.^8–12^).

Metabolomics, or the study of the set of small-molecule metabolites within a biological system, is a powerful approach for identifying endogenous molecules in brain extracellular fluid. Untargeted metabolomics approaches not only detect molecules of known structure that can be identified from standard compounds, they also provide information on metabolites with undetermined structures and functions^13^. Because metabolomics provides functional information about cellular physiology, measuring the brain metabolome may help to clarify how changes in neurochemistry contribute to the generation of sleep and wakefulness^14^. Moreover, metabolomics may provide insights about the functions of sleep. To date, metabolomic studies of sleep and wakefulness have used plasma and urine samples that cannot directly inform about brain changes^15–17^ or brain homogenates that cannot distinguish between intracellular and extracellular metabolites^18^.

New detection methods are now available to analyze brain microdialysis samples with high sensitivity and specificity^19–21^. According to the human metabolome database, 440 compounds have been detected (quantified or not) in the cerebrospinal fluid^22^. How these metabolites relate to metabolites present in the brain extracellular fluid is unknown. The goal of the present study was to characterize changes in the composition of the extracellular fluid that were associated with states of sleep and wakefulness, independent of circadian time or stress caused by sleep deprivation. Ultra performance liquid chromatography—high resolution mass spectrometry (UPLC-HRMS) was used to identify and quantify metabolites in mouse medial prefrontal cortex (mPFC) and primary motor cortex (M1). *In vivo* microdialysis was used to collect samples from mice during electrographically identified states of sleep and wakefulness during times when mice are normally asleep (light period), normally awake (dark period), and during sleep deprivation. We focused on mPFC and M1 because we recently found that whereas noradrenaline levels increased during waking and decreased during sleep in both regions, noradrenaline levels began declining by the end of 6 hours of sleep deprivation only in mPFC. This decline correlated with an increase in low (2–6 Hz) EEG frequencies, a marker of “fatigue”, suggesting that the build-up of sleep pressure during waking may occur faster in mPFC than in M1^23^.

## Results

**Figure 1:**
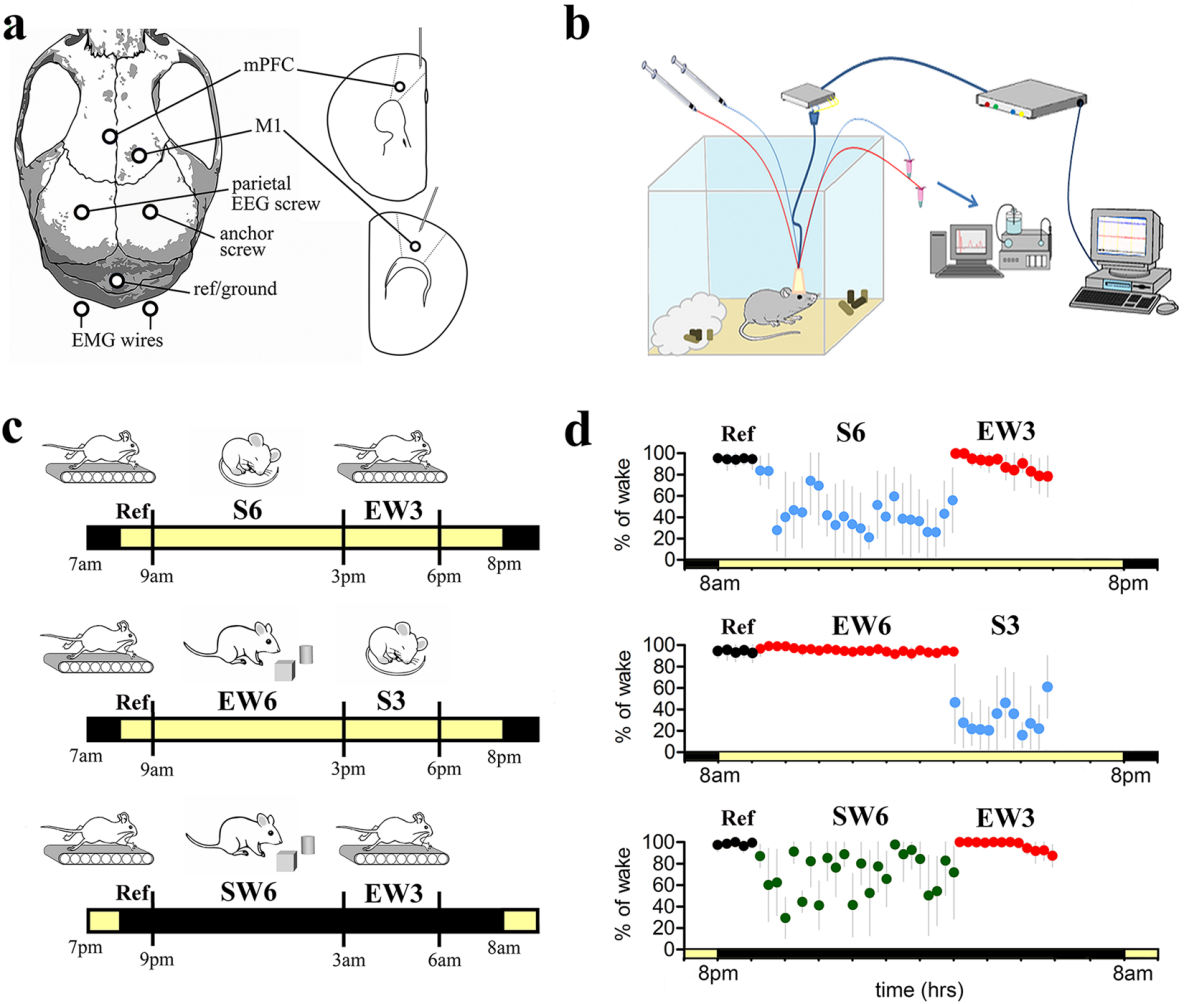
Experimental design. (**a**) Schematic diagram showing the placement of the microdialysis probes, EEG electrodes and EMG wires; (**b**) set-up for EEG recording and microdialysis; (**c**) the 3 experimental groups. (**d**) mean % of wakefulness for each group in 15-min intervals. The 5 time-points in black refer to the end of the first hour and the entire second hour spent by the mice on the treadmill and used as reference (Ref). Blue time points indicate the periods when mice had the opportunity to sleep. Time points in red indicate the period of enforced wakefulness using novel objects. Time points in green show when mice were either spontaneous awake at night or placed on the treadmill for the last 3 hours of the experiment.

Microdialysis samples were collected simultaneously in the mPFC and M1 brain regions of C57BL/6 J (B6) mice over approximately 10 hours in 15-min increments (41 time-points). There were 17 missing samples across all experimental conditions, resulting in a total of 1541 dialysate samples for analysis (Fig. 1a,b). Experiments were performed in three separate groups of animals, all of which were well entrained to the light/dark cycle. All mice spent the first 2 hours awake, slowly moving on a treadmill, and dialysate samples from the end of the first hour and the entire second hour (5 time-points total) were used as reference. Mice were then studied during the following 9 hours in different sleep/wake conditions (Fig. 1c,d). The first 2 groups were studied during the light phase, one group during a sustained period (~6 hours) of sleep followed by 3 hours of enforced wakefulness on the treadmill (S6-EW3), and the other group during 6 hours of wakefulness enforced by exposure to novel objects, followed by 3 hours of sleep (EW6-S3). Thus, these two groups of animals were studied at the same time of day and in the same lighting conditions (lights on) but on average, they spent most of last 9 hours of the experiment in opposite behavioral states (sleep or wake; Fig. 1d). The third group of mice was studied at the opposite circadian time, during the dark phase, and spent the last 9 hours mostly awake, the first 6 hours in their cages, and the last 3 hours on the treadmill (SW6-EW3). The combination of these three groups, including enforced wakefulness during the day and spontaneous wakefulness at night, allowed us to assess the effects of sleep and wakefulness independent of possible confounding factors due to time of day, light exposure, and stimulation (novel objects) used to enforce wakefulness during the day, when mice would spontaneously sleep. From the mPFC and M1, respectively, of each experimental group, the following number of microdialysis samples were collected; 281 and 278 from Group S6-EW3 (n = 7 mice), 246 and 246 from Group EW6-S3 (n = 6), and 244 and 246 from Group SW6-EW3 (n = 6). Following collection, a 10 µL injection of each sample was analyzed by an untargeted metabolomics method using UPLC-HRMS. The time course plots (Fig. 1d) indicate the average amount of time that mice actually spent awake or asleep during the different treatment conditions.

### Heatmap Analysis

**Figure 2:**
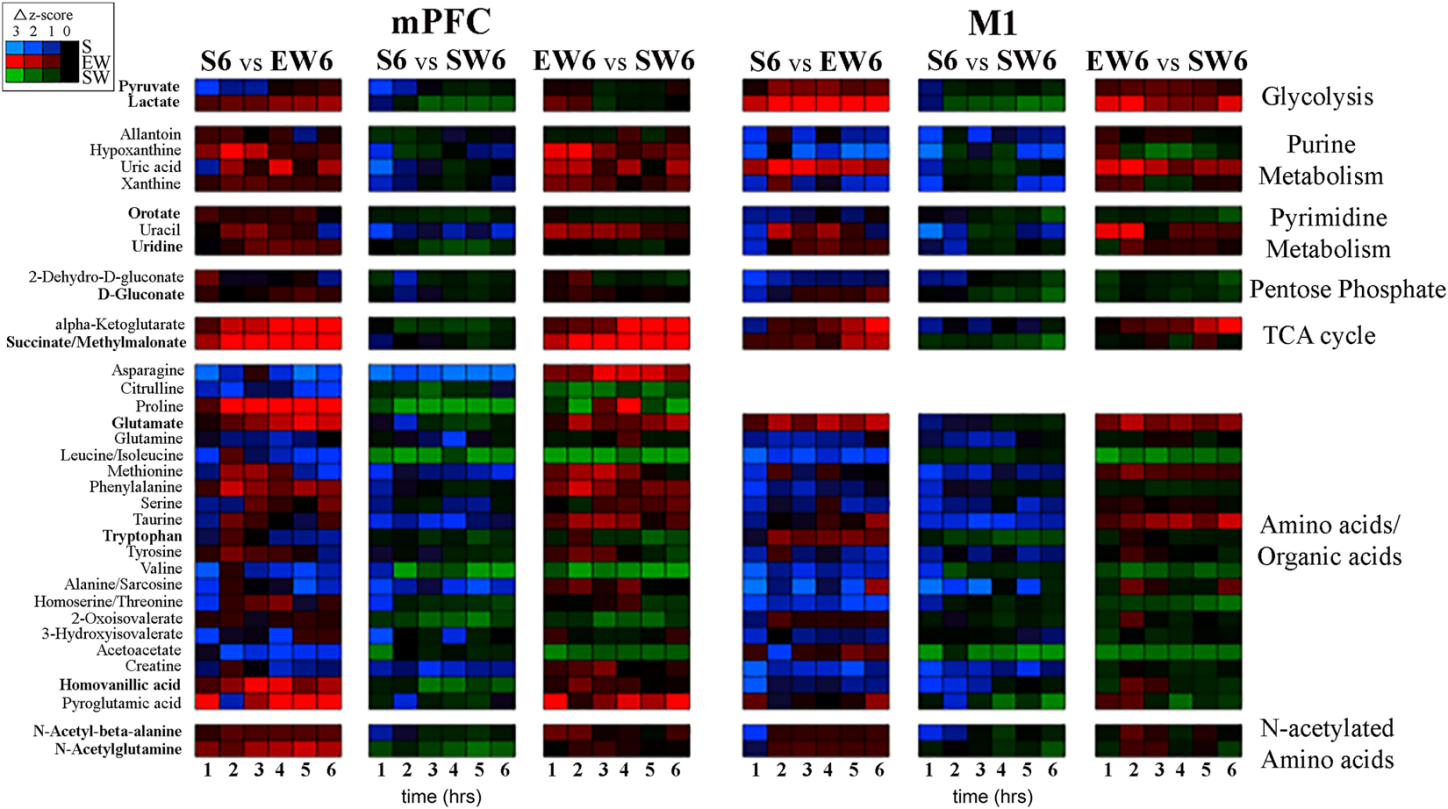
Heatmap analysis. Graphical representation of changes in metabolite levels between microdialysis samples collected from mPFC and M1 expressed as changes in Z score. Each cell represents a 1-hour coarse-grained average ion intensity across the first 6 hours of spontaneous sleep (S6), enforced wakefulness (EW6) or spontaneous wakefulness (SW6). Group comparisons are indicated above each panel. Metabolites (listed at left) are organized by metabolic pathway (listed at right) and listed in bold text when the differences between behavioral states were subsequently identified as statistically significant (LME models). Heatmaps were generated in R with the ggplot2 and colorspace packages.

First, known metabolites were compared in a pairwise fashion between treatment groups using changes in z-score of metabolite intensities as a proxy for relative metabolite concentrations. To visualize these data during the first 6 hours of sleep or wake (S6, EW6, SW6), heatmaps^24^ were constructed that compared metabolite levels across each possible pair of experimental groups in mPFC and separately in M1 (S6 vs. EW6, S6 vs SW6, EW6 vs SW6). This visualization, shown in Fig. 2, conserves the hour-by-hour sampling provided by *in vivo* microdialysis, allowing the identification of metabolic trends between treatment conditions. For example the increases in pyruvate, lactate, orodate, uridine, gluconate, and glutamate during spontaneous wakefulness and/or enforced wakefulness relative to sleep that are described below can be visualized in the heat maps by the presence of more red and/or green cells in the rows corresponding to those analytes.

### Results of LME Models

To promote generalizability of the results, statistical analysis was restricted to metabolites that were observed at least in 3 mice per treatment condition. This selection criterion was applied separately to each brain region and provided a list of 33 known metabolites common to both mPFC and M1, and 3 metabolites that were unique to mPFC (36 metabolites total; see Methods for details). Three linear mixed effect models were applied to each metabolite. The first model focused on the first 6 hours, and the levels of each compound were compared “across groups” of mice that spent most of that time in different behavioral states, either sleep, or enforced wakefulness or spontaneous wakefulness (S6, EW6, SW6). The other two models were “within group” models, i.e. they compared the levels of each metabolite during 9 consecutive hours in the same mouse, either during spontaneous sleep followed by enforced wakefulness (S6-EW3), or during enforced wakefulness followed by spontaneous sleep (EW6-S3). To be considered as state-specific, a known metabolite (or unidentified spectral features, see below) was required to meet the following criteria: 1) show a statistically significant change in at least one model (p < 0.05); and 2) show biologically consistent changes in the other models, even if those changes did not reach significance. For example, a compound that showed a significant increase in the wake groups (SW6, EW6) relative to the sleep group (S6) but no increase in the same mouse during the wake period after sleep (S6-EW3) and no decline in the same mouse during the sleep period after wakefulness (EW6-S3) was not considered “state-specific”.

**Figure 3:**
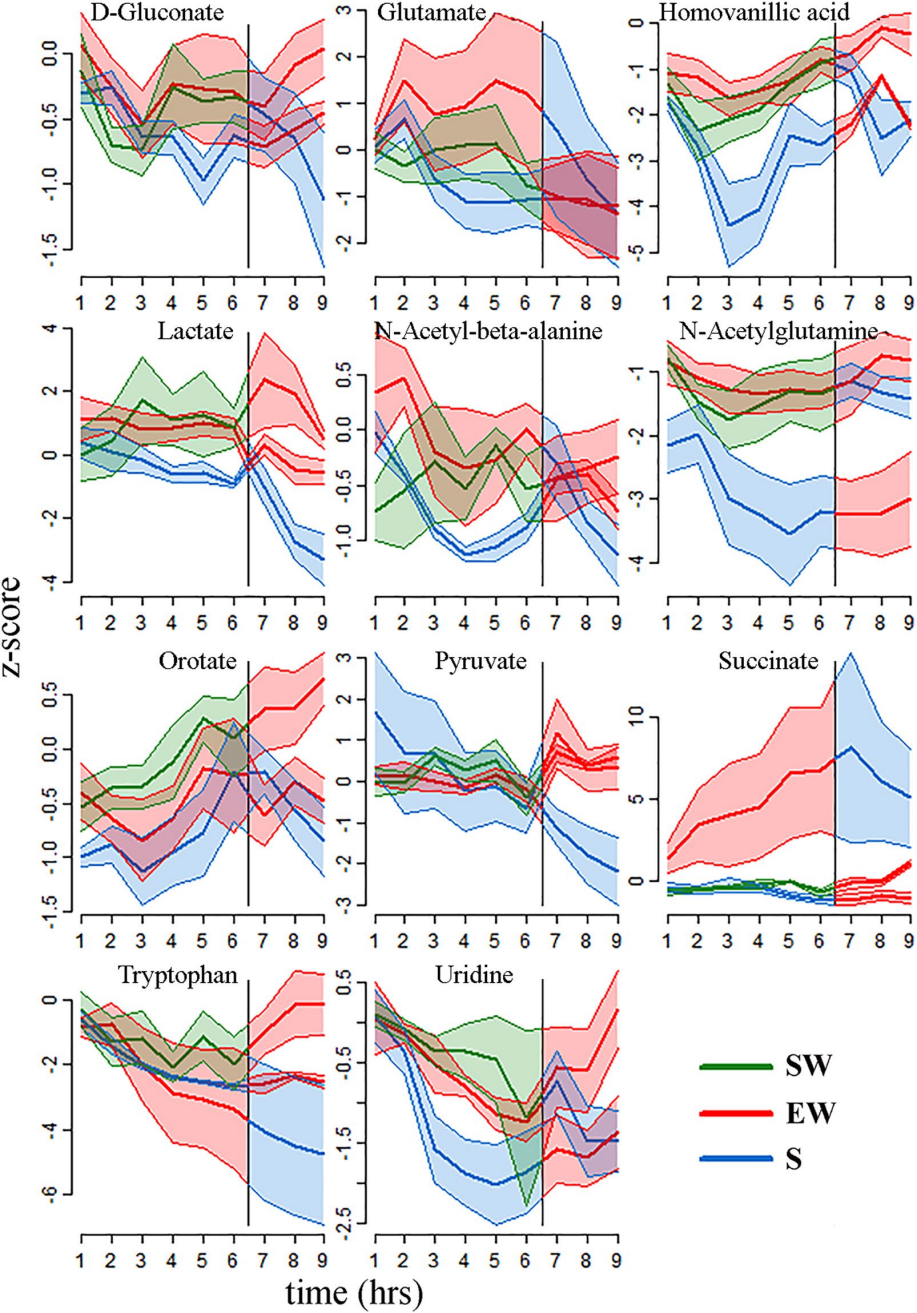
Time course of z-score changes for the 11 metabolites with significant and consistent differences between behavioral states in mPFC (mean ± sem). In each plot the vertical line indicates a behavioral state transition at hour 6 (e.g, from S6 to EW3). Typically, the levels of these metabolites were decreasing during sleep, and either increasing or constant during the two wakefulness conditions. Abbreviations: mPRF (medial prefrontal cortex); SW, spontaneous wake; EW enforced wake; S, spontaneous sleep.

**Figure 4:**
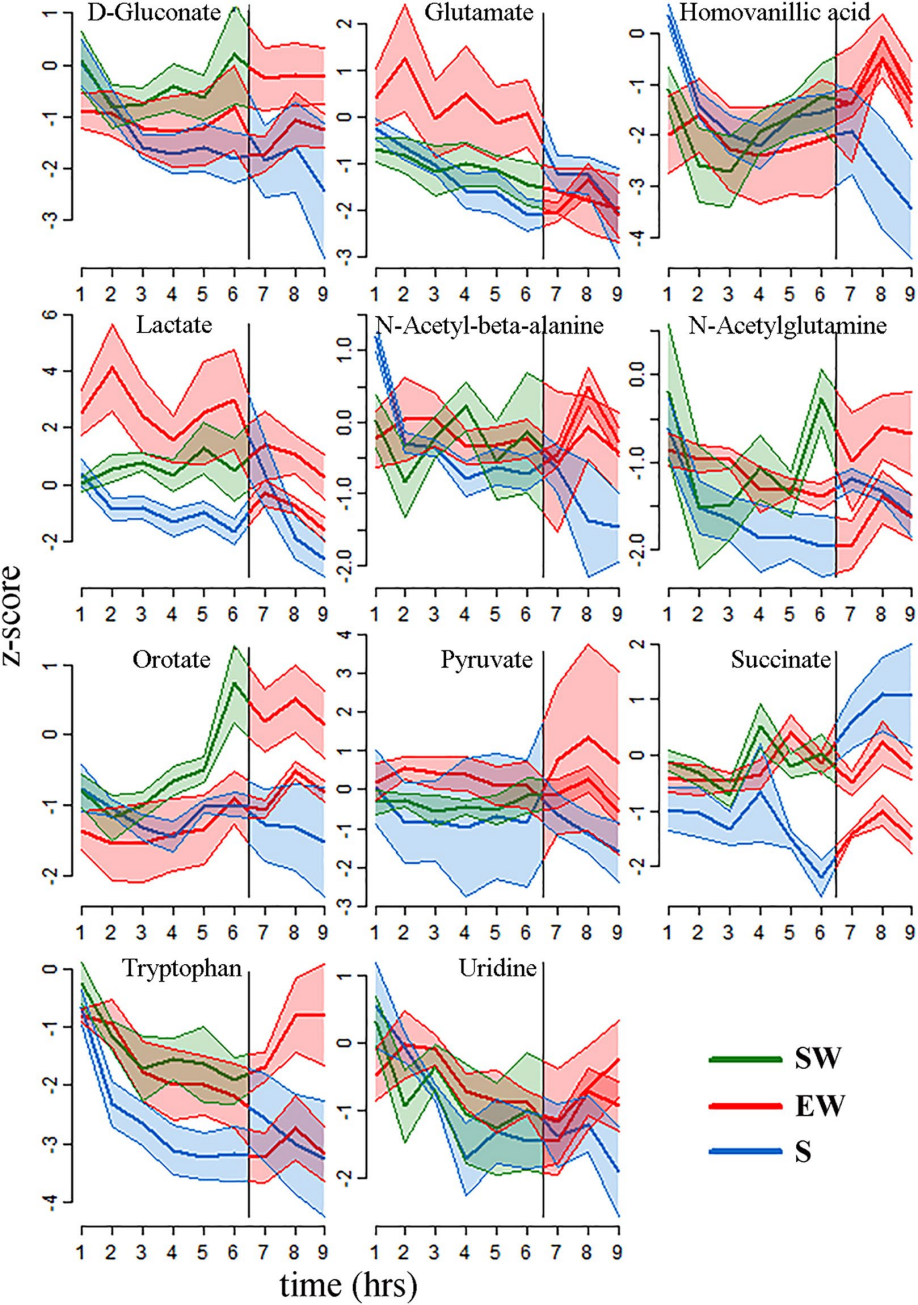
Time course of z-scored changes for the 11 metabolites with significant and consistent differences between behavioral states in M1 (mean ± sem). In each plot the vertical line indicates a behavioral state transition at hour 6. Similar to mPFC, metabolite levels tended to decrease during sleep, and either increased or remained constant during the two wakefulness conditions. Abbreviations: M1, primary motor cortex; SW, spontaneous wake; EW, enforced wake; S, spontaneous sleep.

Table 1 lists the 11 known metabolites that showed changes with sleep and wakefulness according to these strict criteria. These 11 metabolites represented approximately 30% of the metabolites consistently detected in our samples (33 in M1, 36 in mPFC). All of the 11 analytes reported in Table 1 decreased throughout the sleep period relative to both wake conditions (Figs 3 and 4). Among the 33 comparisons presented, in 31 we identified a significant behavioral state * time interaction, with the differences between behavioral states being more pronounced at the end of the experiment than at the beginning. For the remaining 2 comparisons, the differences between the sleep and wake conditions were consistent for the duration of the experiments (Table 1). One analyte (homovanillic acid) showed a significant condition by brain region interaction, with state-specific changes occurring primarily in mPFC. Several other metabolites, including homoserine/threonine, uracil, and valine, showed highly significant temporal effects in all three behavioral states, with levels decreasing from hour 1 to hour 9. This profile likely reflects some form of “depletion” during prolonged sampling, and these analytes are not further discussed.

**Table 1:** Significance levels of the 11 known metabolites with consistent differences between behavioral states.

Metabolite	Between Groups (S6-SW6-EW6)	Within Group (S6-EW3)	Within Group (EW6-S3)
D-Gluconate	0.0032(**)	0.0007(***)	0.1291
Glutamate	0.0253(*)	0.0965	0.0037(**)
Homovanillic acid	0.0051(**)	0.0125(*)	0.0000(***)
Lactate	0.0020(**)	0.0386(*)	0.0000(***)
N-acetyl-beta-alanine	0.0013(**)	0.0264(*)	0.0169(*)
N-acetyl-glutamine	0.0894	0.0336(*)	0.0554
Orotate	0.1067	0.0006(***)	0.0057(**)
Pyruvate	0.8409	0.0012(**)	0.0040(**)
Succinate/Methylmalonate	0.0000(***)	0.2814	0.3415
Tryptophan	0.4869	0.0000(***)	0.4829
Uridine	0.0363(*)	0.0000(***)	0.6407

The p-values generally reflect a behavioral state by time interaction, with the sleep conditions decreasing while the wake conditions remained constant (or increase). Pyruvate (S6-SW6-EW6) and orotate (S6-EW3) were the only two exceptions, in that the reported p-values represent a main effect of state and no time interaction). (*)p < 0.05, (**)p < 0.01, (***)p < 0.001. Abbreviations: S, sleep; SW, spontaneous wakefulness; EW, enforced wakefulness.

**Figure 5:**
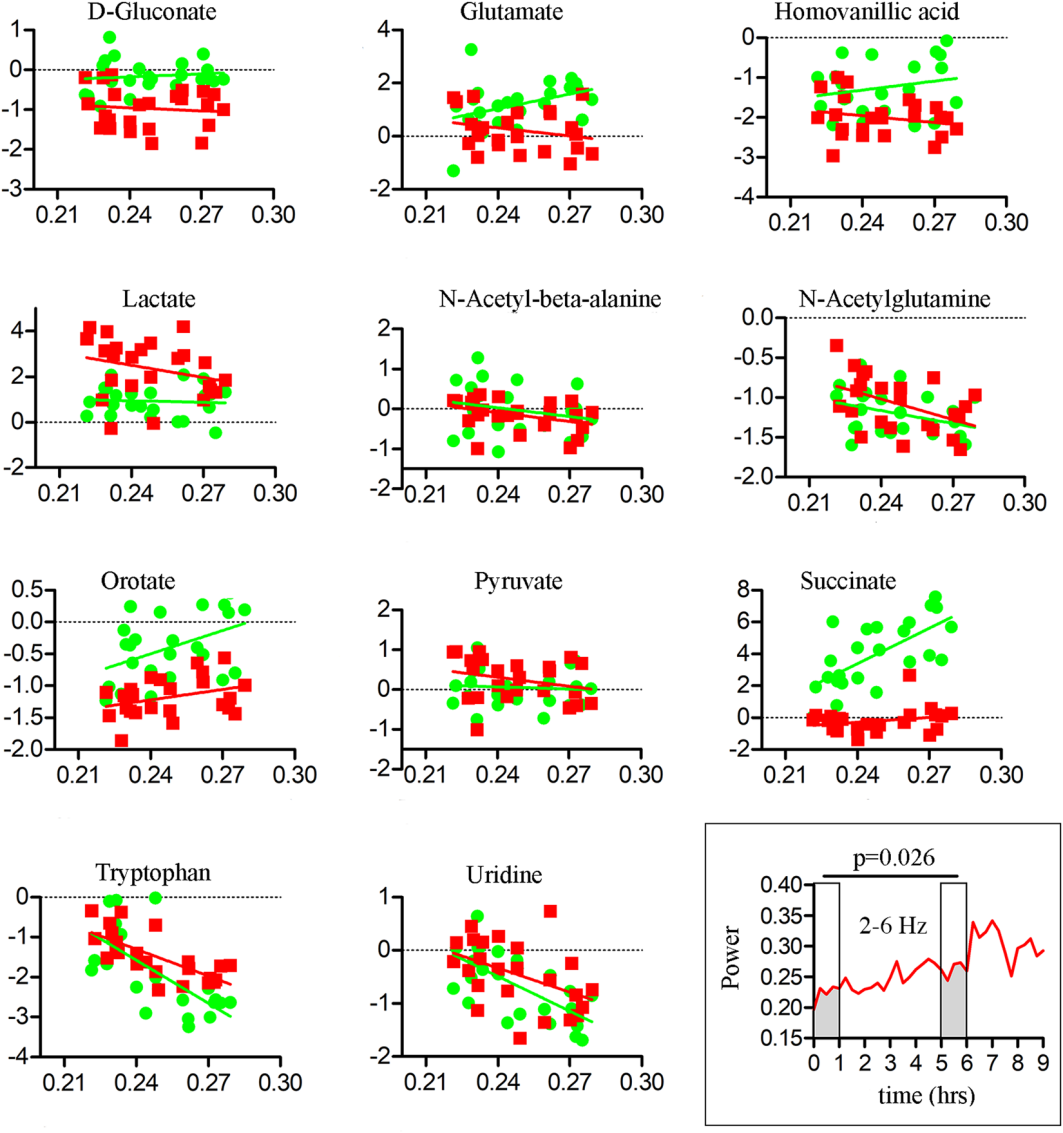
Correlations between levels of each compound (log transformed; y axis) and 2–6 Hz EEG activity during wakefulness (x axis). Data and regression lines are depicted in green for mPFC and red for M1. The inset (bottom right) shows the averaged time course (n = 6 mice) of 2–6 Hz activity during the EW6-S3 experiment. Note the progressive increase in 2–6 Hz activity in the course of EW. Abbreviations: mPFC, medial prefrontal cortex; M1, primary motor cortex; EW, enforced wakefulness; S, sleep.

We then tested whether changes in the levels of these 11 metabolites were correlated with changes in the EEG power spectrum. The analyses focused on low frequency (2–6 Hz) activity during wakefulness and slow wave activity (SWA, 0.5–4 Hz) during NREM sleep, two markers of sleep pressure that increase during sleep deprivation and subsequent recovery sleep, respectively^23^. Consistent with previous findings in humans and rodents, low frequency activity increased significantly across the course of the 6 hours of sleep deprivation (EW6, Fig. 5 inset). This increase correlated negatively with the increase of tryptophan and uridine in mPFC, and of tryptophan in M1 (after correcting for multiple comparisons; Fig. 5, Table 2). Succinate/Methylmalonate was the only metabolite positively correlated with 2–6 Hz activity, and only in mPFC (Fig. 5, Table 2).

**Table 2:** Pearson’s coefficients and p-values between log-values for levels of each compound and 2–6 Hz activity or SWA.

Metabolite	2–6 Hz activity				SWA (0.5–4 Hz)			
	*mPFC*		*M1*		*mPFC*		*M1*	
	r	p		p	r	p	r	p
D-gluconate	0.1205	0.5748	−0.0951	0.6584	0.4403	0.1520	0.4396	0.1527
Glutamate	0.3861	0.0624	−0.2459	0.2468	0.8016	**0.0017**	0.4535	0.1387
Homovanillic Acid	0.2153	0.3124	−0.2152	0.3125	0.6874	**0.0135**	0.6839	**0.0142**
Lactate	−0.0657	0.7605	−0.2680	0.2054	0.8380	**0.0007**	0.6731	**0.0164**
N-acetyl-beta-alanine	−0.2293	0.2812	−0.3562	0.0875	0.8273	**0.0009**	0.6522	**0.0215**
N-acetyl-glutamine	−0.3561	0.0877	−0.4672	0.0213	0.3647	0.2437	0.7362	**0.0063**
Orotate	0.4405	0.0312	0.3337	0.1110	0.6318	**0.0275**	0.4993	0.0984
Pyruvate	−0.0726	0.7362	−0.2643	0.2121	0.8331	**0.0008**	0.6877	**0.0134**
Succinate/Methylmalonate	0.6722	0.0003	**0.2451**	0.2483	0.6908	**0.0129**	−0.3926	0.2069
Tryptophan	−0.6818	0.0002	**−0.7175**	**0.0001**	0.7250	**0.0076**	0.8837	**0.0001**
Uridine	−0.6602	0.0004	**−0.4474**	0.0284	0.6901	**0.0130**	0.5192	0.0837

Values in bold are the ones surviving the correction for multiple comparison (Benjamini-Hochberg, 1995).

**Figure 6:**
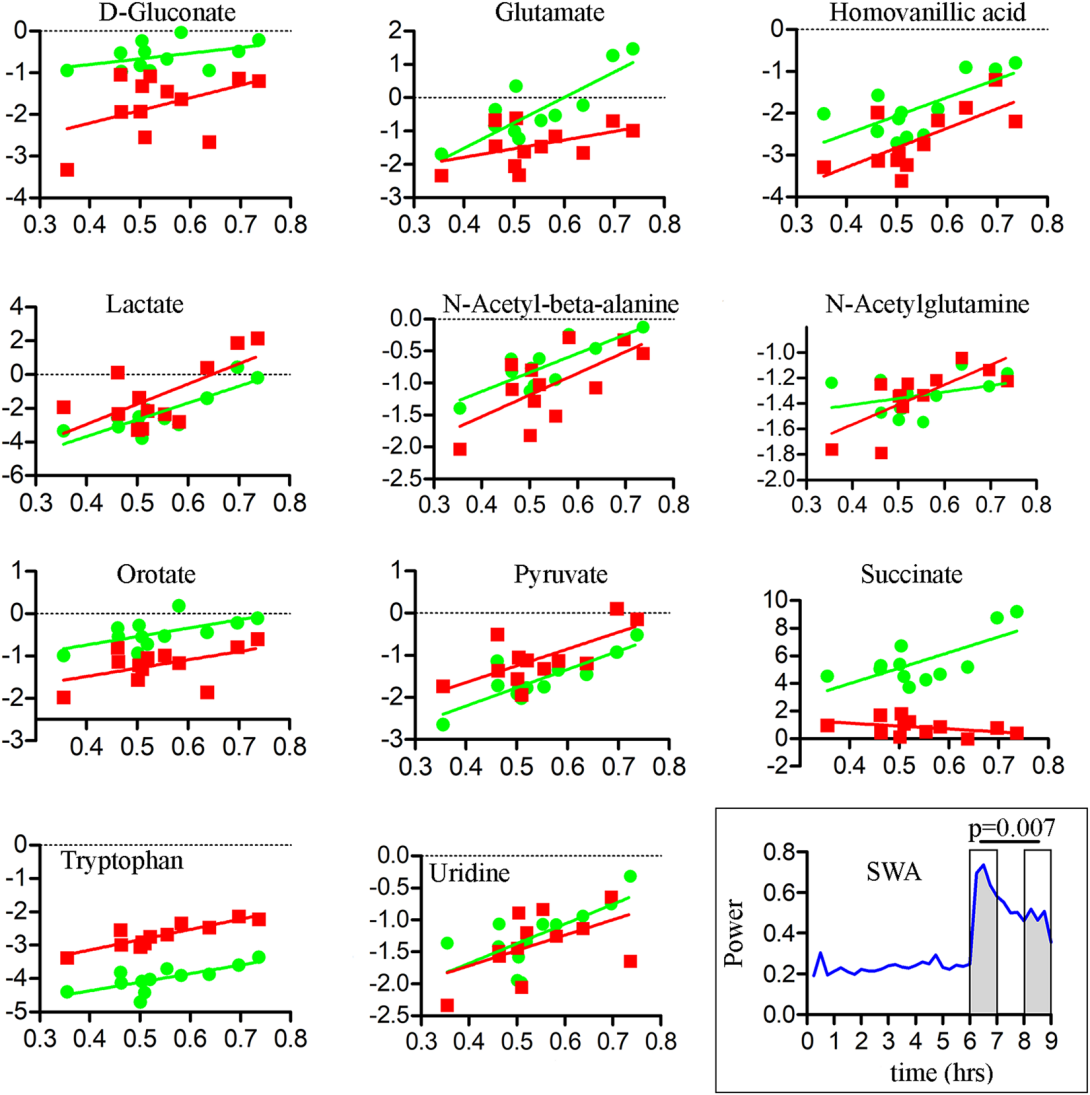
Correlations between levels of each compound (log transformed; y axis) and SWA during recovery sleep (x axis). Data and regression lines are depicted in green for mPFC and red for M1. The inset (bottom right) shows the averaged SWA time course (n = 6 mice) during the EW6-S3 experiment. Note the progressive SWA decline in the course of the 3 hours of recovery sleep (hours 6–9). Abbreviations: mPFC, medial prefrontal cortex; M1, primary motor cortex; SWA, slow wave activity; EW, enforced wakefulness; S, sleep.

As expected SWA was high at sleep onset and declined significantly in the course of recovery sleep following sleep deprivation (Fig. 6 inset). For most metabolites the decline during sleep was positively correlated with the decline in SWA, especially in mPFC (Fig. 6, Table 2). There were no correlations with gamma activity (40–100 Hz) during wake (not shown).

An additional LME model was used to compare the concentration of metabolites during 3 different sleep periods: “early sleep” and “late sleep” were defined as the first 3 and the last 3 hours of the S6 experiment, respectively, and “recovery” indicated the 3 hours of recovery sleep after the EW6 experiment (see Methods for details). Specifically, we asked whether the trend for a metabolite concentration to decrease was different among early, late and recovery sleep. The results are summarized in Table 3 and demonstrated a significant interaction between sleep condition and time in all compounds and typically in both regions, with two exceptions (pyruvate in mPFC and glutamate in M1). Other than succinate, all significant interactions followed the same general pattern, with metabolite concentration decreasing faster during early sleep and recovery sleep than during late sleep, consistent with higher sleep pressure in the first two conditions. To further distinguish early sleep from recovery sleep we then performed post-hoc tests to classify each interaction into one of 3 possibilities, depending on whether the rate of concentration decrease did not differ between early sleep and recovery sleep (type 1), was faster in recovery sleep than in early sleep (type 2), or vice versa (type 3). For only 2 metabolites, glutamate and lactate, the concentration decreased faster during recovery sleep than during early sleep.

**Table 3:** Comparisons between early and late periods of spontaneous sleep and recovery sleep.

Metabolite	Region	p-value	Pattern
D-Gluconate	mPFC	0.0385(*)	Type1
	M1	0.0007(***)	Type3
Glutamate	mPFC	0.005(**)	Type2
	M1	0.754	
Homovanillic Acid	mPFC	1.7e-8(***)	Type3
	M1	8.7e-15(***)	Type3
Lactate	mPFC	2.9e-6(***)	Type2
	M1	7.8e-5(***)	Type2
N-Acetyl-beta-alanine	mPFC	2.0e-5(***)	Type1
	M1	0.0001(***)	Type3
N-Acetylglutamine	mPFC	0.007(**)	Type1
	M1	1.5e-6(***)	Type3
Orotate	mPFC	1.2e-5(***)	Type1
	M1	0.003(**)	Type1
Pyruvate	mPFC	0.2527	
	M1	0.002(**)	Type1
Succinate/Methylmalonate	mPFC	0.025(*)	other
	M1	0.018(*)	other
Tryptophan	mPFC	4.8e-5(***)	Type3
	M1	5.3e-8(***)	Type3
Uridine	mPFC	0.0014(**)	Type3
	M1	0.0007(***)	Type1

Significance of condition * time interaction for each of the 11 significant compounds, in each region. For this analysis Time is a continuous covariate (linear). Condition is either “Early” (the first 3 hours of the S6 condition), “Late” (the last 3 hours of the S6 experiment) or Recovery (the 3 hours of sleep from the EW6-S3 experiment). Type 1: no significant difference between the conditions; Type 2: recovery sleep decreases significantly faster than early sleep; Type 3: early sleep decreases significantly faster than recovery sleep. (*)p < 0.05, (**)p < 0.01, (***)p < 0.001.

Finally, 17 of the unidentified spectral features of the UPLC-HRMS spectra were reliably detected in the majority of animals in each of the 3 experimental groups and changed with sleep and wakefulness according to the same strict criteria discussed above for the known metabolites, including 16 that were increased in wake and 1 that was increased in sleep. Table 4 provides the mass and retention times for these unknown features as well as the results of the statistical analyses.

**Table 4:** Significance levels of 17 unknown features with consistent differences between behavioral states (p-values).

Mass	Retention Time	Between Groups (S6-SW6-EW6)	Within Group (S6-EW3)	Within Group (EW6-S3)
146.96404	8.24	0.1150	0.0030(**)	0.0000(***)
173.00636	5.35	0.0104(*)	0.0000(***)	0.0387(*)
263.04372	3.74	0.7308	0.0022(**)	0.0171(*)
90.02694	6.49	0.1210	0.0000(***)	0.0002(***)
125.00052	6.46	0.0004(***)	0.0000(***)	0.1352
169.01140	8.22	0.0284(*)	0.0710	0.0022(**)
88.01126	8.26	0.0352(*)	0.0497(*)	0.0000(***)
89.02361	1.21	0.0231(*)	0.6241	0.1312
126.99327	6.59	0.0074(**)	0.0110(*)	0.0118(*)
87.20528	5.19	0.0010(**)	0.0365(*)	0.0437(*)
244.99770	8.25	0.0011(**)	0.0037(**)	0.0000(***)
157.01139	5.31	0.0012(**)	0.0000(***)	0.0414(*)
206.01253	12.07	0.5829	0.9685	0.0000(***)
203.93613	12.72	0.1758	0.0250(*)	0.0000(***)
209.02997	5.27	0.0236(*)	0.0004(***)	0.0017(**)
473.07362	1.17	0.0001(***)	0.0014(**)	0.0050(**)
135.02959	12.69	0.0069(**)	0.0006(***)	0.0000(***)

The p-values generally reflect either a behavioral state by time interaction, with the levels generally decreasing in the sleep conditions and the levels remaining constant or increasing during the wakefulness conditions, or a main effect of behavioral state with lower values during sleep than the wake conditions. One exception is the features with mass of approximately 473, which displayed a spike at the onset of sleep that then decayed over time back to baseline levels. (*)p < 0.05, (**)p < 0.01, (***)p < 0.001.

### Classification Analyses

**Figure 7:**
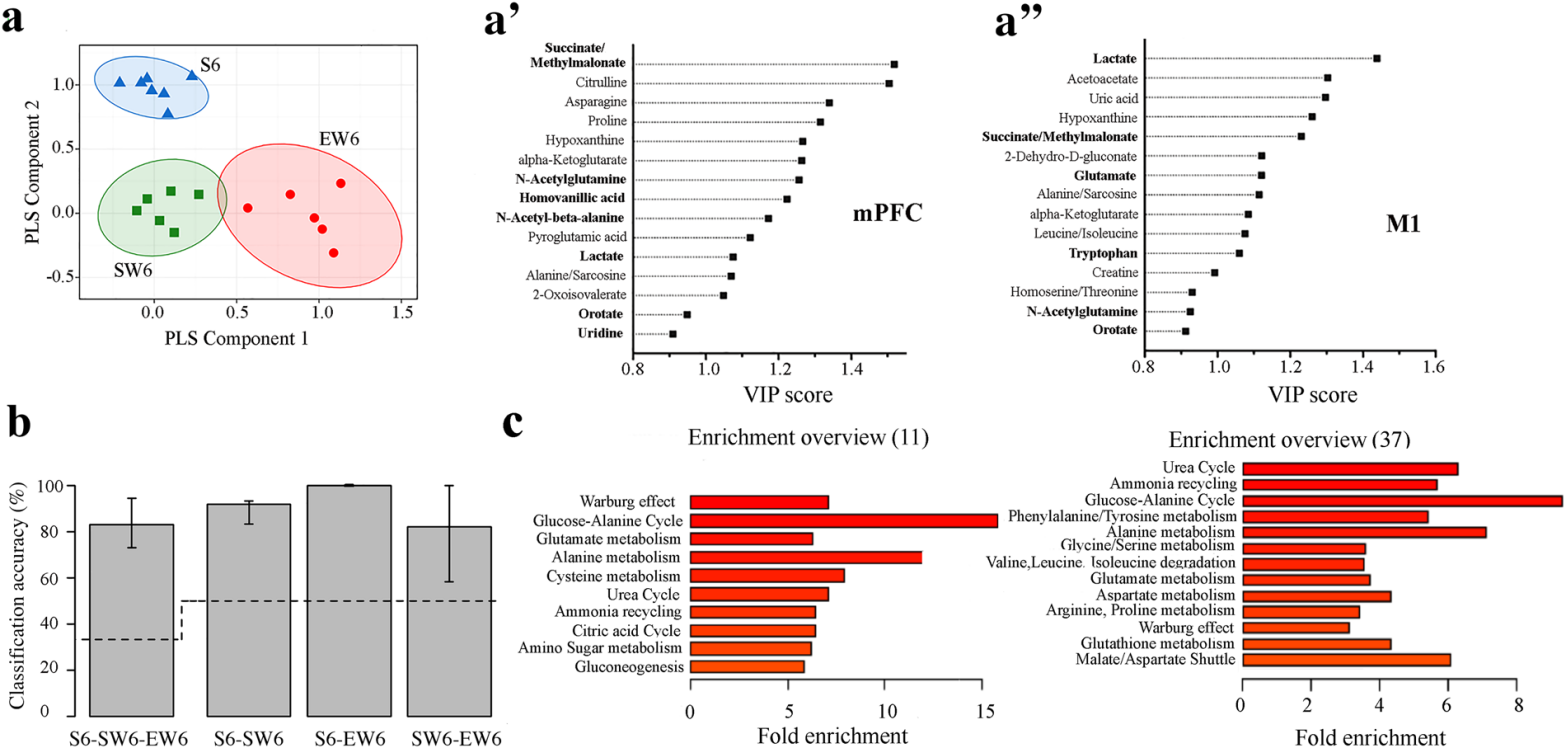
PLSDA and associated VIP classification model. By combining the mPFC and M1 analyses, data from S6, EW6, and SW6 were used in the PLSDA to determine the overall variation between treatment conditions. (**a**) PLSDA plot showing significant separation of EW6 from S6 and SW6 in Component 1, and additional separation of S6 from SW6 and EW6 in Component 2. Each symbol indicates data from one mouse. Ellipses represent a confidence interval of 95%. VIP score plots for mPFC (a’) and M1 (a”), showing the 15 metabolites with the highest VIP scores. The bold text indicates the metabolites identified by LME analyses. (**b**) Classification accuracy of the logistic regression with ℓ2 regularization penalty. Error bars represent a non-parametric 95% confidence interval for the true accuracy estimated from the cross-validation data. The lower end of the confidence interval is above the chance line (dashed line) for all 4 comparisons. Abbreviations: mPFC, medial prefrontal cortex; M1, primary motor cortex; S, sleep; EW, enforced wakefulness; SW, spontaneous wakefulness; PLSDA (partial least squares discriminate analyses; VIP, variable importance of projection; LME, linear mixed effects. (**c**) Metabolite set enrichment analysis using the 11 metabolites differentially expressed between sleep and wake (left) and all 37 metabolites (right). Pathways with >2-fold enrichment (all p < 0.05) are shown.

Partial least squares discriminant analyses (PLSDA) were used to determine whether the metabolic profiles during the first 6 hours of sleep, enforced wakefulness, and spontaneous wakefulness could distinguish between the 3 behavioral states. All known metabolites consistently detected in our samples (33 in M1, 36 in mPFC) were used for this analysis. PLSDA is a commonly used statistical approach in metabolomics because it is properly suited for an uneven design matrix, in which the number of dependent variables (detected metabolites) is larger than the number of independent variables (number of samples). Indeed, the metabolite profile from all 3 experimental groups (S6, EW6, SW6) was sufficient to classify the state of arousal using data from both mPFC and M1. The PLSDA model perfectly classified the mice into their respective experimental conditions. As shown in Fig. 7a plotting the PLSDA score values of PLS Component 1 and PLS Component 2 demonstrated visible clustering and clear separation between the 3 experimental groups. Additionally, PLSDA identified the metabolites that drive the separation among conditions by ascribing a variable importance of projection score (VIP). As the weighted sum of the squared correlations between PLSDA components and the original variables, these values represent the percentage of variation explained by the component in the model. Generally, a VIP greater than 1.0 is considered most significant (i.e., metabolites with VIP >1 strongly contribute to the observed differences among groups). The 15 metabolites with the highest VIP scores are listed in Fig. 7a’ (mPFC) and 7a” (M1).

With so many explanatory variables and a relatively small number of mice, overfitting the PLSDA model was a concern. To better understand how the results will generalize, a K-fold stratified cross-validation procedure was used to estimate the accuracy of the classifier and statistical significance was assessed using non-parametric permutations tests. The cross-validation analysis resulted in an estimated accuracy of 76.4%, which was significantly greater than chance (p < 0.05). We then explored alternative classification models to see if we could improve on the accuracy of PLSDA. The best accuracy was achieved using a logistic regression classifier with an ℓ2 regularization penalty with sparsity parameter C = 0.001 (smaller values of C restrict the model and promote more sparsity). For this classifier the cross-validation procedure resulted in an estimated accuracy of 83%, which was significantly greater than chance (p < 0.001). Further exploring the ability for this classifier to pair-wise discriminate between conditions, we found that the sleep condition was distinguished from the two wake conditions with greater accuracy (S6 vs. SW6 accuracy 91.8%, p < 0.01; S6 vs. EW6 accuracy 100%, p < 0.01) than the two wake conditions could be distinguished from each other (SW6 vs. EW6 accuracy 82.1%, p = 0.003; Fig. 7b). This is consistent with the visualization of the PLSDA model (Fig. 7a), in which the clusters corresponding to the 2 wake conditions are closer to each other than they are to the sleep cluster.

Finally, we performed metabolite set enrichment analysis (MSEA;^25^) using the 11 metabolites identified as differentially expressed between sleep and wake, as well as the entire set of 37 metabolites expressed in most mice in at least one brain area (36 peaks, corresponding to 37 metabolites because succinate/methylmalonate could not be resolved in two separate peaks). The majority of the enriched pathways (>2-fold enrichment, in all cases p < 0.05) were identified using either 37 or 11 metabolites, although some differences were also present (Fig. 7c).

## Discussion

Many previous microdialysis studies measured neurotransmitters, neuropeptides and select metabolites in the rodent brain across the sleep/wake cycle. In some experiments the high temporal resolution of biosensors was used to detect rapid changes in cortical levels of glutamate, lactate and glucose during wakefulness, NREM sleep and REM sleep^26–30^. In these reports, however, one or at most a few analytes were measured^31,32^. A recent study applied liquid chromatography and online mass spectrometry to the mouse prefrontal cortex to test a metabolomics platform of almost 300 metabolites, of which approximately 120 were identified in the extracellular fluid^33^, but sleep/wake changes were not studied. Thus, to our knowledge, this is the first study providing a broader view of metabolome changes in the cortical extracellular space across the sleep/wake cycle and after sleep deprivation.

More than 30 small molecules in more than 80% of mice were identified. According to the LME model analysis, 11 of these known metabolites were present at significantly higher levels after wakefulness, in most cases after both spontaneous and enforced wakefulness and in both cortical regions, while no metabolite was detected at higher levels after sleep. A similar trend was present among the differentially regulated unidentified spectral features, with 16 upregulated in wakefulness and 1 in sleep. This “imbalance” between wake-related and sleep-related compounds is not new. Among the hundreds of transcripts modulated by sleep and wakefulness in neurons, astrocytes, and oligodendrocytes of rat and mouse cortex, the majority were transcripts whose expression was higher in both spontaneous and enforced wakefulness than in sleep^1,6^, reflecting either increased transcription and/or decreased degradation of mRNAs during waking. In the current study, higher wake levels of analytes could reflect increased synthesis, decreased utilization, and/or decreased elimination by the glymphatic system that drives exchange of interstitial fluid with cerebrospinal fluid^34,35^. Wake-related metabolites reflect different signaling pathways and cellular processes and many, but not all, were already known to be differentially expressed across behavioral states. The potential relevance of each metabolite to states of sleep and wakefulness is briefly considered below.

Two energy metabolites were upregulated in wake relative to sleep, lactate and pyruvate, consistent with previous studies in rats and mice^27,28,36–38^. Astrocytes have strong glycolytic activity and produce lactate from glucose and glycogen via aerobic glycolysis while neurons, which have high oxidative capacity, mainly convert lactate into pyruvate to be used in the tricarboxylic acid cycle in the mitochondria^39^. Excitatory glutamatergic activity, which is higher in wakefulness than in sleep^26^, leads to increased astrocytic uptake of glucose, followed by an increase in the production of lactate, its release in the extracellular space, and uptake by neurons^39^. However, neurons can also directly uptake glucose and do not rely exclusively on astrocytes to produce pyruvate^39,40^. Thus, why astrocytes should release more lactate in response to neuronal activation is not obvious, at least if one assumes that lactate acts mainly as an energy substrate. On the other hand, aerobic glycolysis is stimulated in conditions of synaptic growth, is blocked by inhibition of the noradrenergic system^41^, and lactate released from astrocytes excites locus coeruleus neurons and increases the release of noradrenaline^42^. Lactate may also serve as a volume transmitter and the G protein coupled lactate receptor GPR81 is concentrated in the synaptic membranes of excitatory synapses^43^. Thus, lactate upregulation during wake may reflect increased synaptic growth and potentiation more than increased energy demand^39,44^. The latter could be met via increases in oxidative phosphorylation, and indeed mitochondrial enzymes of the respiratory chain are upregulated during waking^45^. Of note, the sleep-related drop in lactate levels was faster during recovery sleep than during early spontaneous sleep, and correlated with the decline in SWA, consistent with previous findings^27^. Overall, these results suggest that the regulation of sleep need may be linked to glycolytic processes^46^ and astrocytic functions^47–49^.

The gluconate shunt is a less known metabolic pathway that can facilitate the breakdown of glucose. It has been studied mainly in plants and microorganisms but key enzymes of the shunt, glucose dehydrogenase and gluconate kinase, have been identified in mammals^50^. In this pathway glucose is oxidized by glucose dehydrogenase to gluconic acid (or gluconate). Gluconate is then phosphorylated by gluconate kinase to produce 6-phosphogluconate, the second intermediate of the pentose phosphate pathway, thus bypassing the first rate-limiting step of this pathway^51^. The main function of the pentose phosphate shunt is anabolic rather than catabolic, producing pentose sugars for the synthesis of nucleic acids and amino acids. Thus, the increase in gluconate levels during wakefulness may signal the need for growth, rather than for energy.

The *de novo* pyrimidine pathway provides critical components for the synthesis of DNA, RNA, sugars, glycogen as well as phosphatidylcholine and phosphatidylethanolamine, the major phospholipids of the cellular membranes^52,53^. The levels of two intermediates in this pathway, orotate and uridine, were upregulated with wakefulness. Previous studies also found increased extracellular uridine content after neuronal depolarization^54^. Most uridine in the brain comes from the plasma. However, all genes responsible for the synthesis of pyrimidines are expressed at high levels in the cerebral cortex and other brain regions^52^, suggesting that the brain’s need for pyrimidines may be met also by *in situ* production. After its uptake by neurons and glia, uridine can be phosphorylated to UTP to serve as precursor for RNA and DNA synthesis, or it can be degraded glycolitically to maintain ATP levels^55^. UTP is also released in the extracellular space where is converted back to uridine or has neurotrophic effects after binding P2Y receptors^54^. Uridine also promotes neurite outgrowth and spinogenesis, but whether these effects depend on a specific “uridine receptor” remains unclear^54,56^. Thus, like lactate and gluconate, increased orotate and uridine levels during wakefulness may signal the need for the synthesis of many essential components, from nucleic acids to cellular and synaptic membranes. Of note, uridine was one of the active components of the sleep promoting substance purified from the brainstem of sleep deprived rats, and intracerebral infusion of uridine promotes sleep^56^, suggesting that extracellular high uridine levels may reflect increased sleep need. Consistent with this idea, the decline of uridine levels during recovery sleep was correlated with the SWA decline. Intriguingly, the increase of uridine in the course of 6 hours of sleep deprivation was negatively, rather than positively, correlated with the 2–6 Hz EEG activity. This negative correlation was strongest in mPFC but present also in M1. In both humans and rodents the increase in low frequency activity during extended wake reflects the tendency of some cortical neurons to stop firing and go “off line”, as during NREM sleep^57,58^. Thus, we speculate that uridine levels reflect sustained firing of cortical neurons.

Succinate/Methylmalonate could not be resolved in separate peaks. Succinate is an intermediate of the tricarboxylic acid cycle and methylmalonate (MMA) is converted to succinyl coenzyme-A by an enzymatic reaction that uses vitamin B12 (cobalamin) as a cofactor. High levels of MMA, due to vitamin B12 deficiency or some genetic disorders, can inhibit the activity of the mitochondrial respiratory chain complex by interfering with the mitochondrial uptake of succinate^59,60^. Elevated serum MMA levels have been reported in patients with idiopathic Parkinson’s disease and may be one of the biomarkers for this pathology^61^.

The wake-related increase in extracellular levels of glutamate confirms and extends previous findings in rat^26^ and mouse^28^ cerebral cortex, and is consistent with overall increased glutamatergic neurotransmission during wakefulness relative to sleep. The state-specific changes in glutamate measured here also are consistent with evidence that glutamate in pontine reticular formation of rat also decreases during sleep^21^. Moreover, we found that the progressive decrease in cortical glutamate concentration was faster during recovery sleep than during early spontaneous sleep, consistent with previous evidence that the sleep-dependent decline of glutamate correlates with SWA^26^. As for lactate, this result suggests that the rate of glutamate changes may reflect sleep pressure, although circadian time, the stimulation used to enforce wake, or the “exercise” on the moving wheel during baseline may have contributed to the difference between spontaneous sleep and sleep following sleep deprivation.

Two other metabolites upregulated during wakefulness, homovanillic acid (HVA) and tryptophan, reflect the activity of well-known groups of wake-active neurons. HVA is one of the major dopamine (DA) metabolites and a valid indicator of DA turnover. DA extracellular levels in the mPFC are increased by novelty and higher during wakefulness than during sleep^31,62^, consistent with the fact that DA neurons in the ventral tegmental area, which project to mPFC, are more active during wakefulness than during sleep and promote arousal^63,64^. Tryptophan is an essential amino acid and the precursor of the neurotransmitter serotonin. Like DA neurons in the VTA, serotonin neurons in the dorsal raphe are more active during wakefulness than during sleep and extracellular levels of serotonin are higher in wakefulness than in sleep in different brain regions, including prefrontal cortex^65^.

N-Acetyl-beta-alanine is an endogenous β amino acid that can be converted to acetate and β alanine by N-acetyl-β alanine dehydrogenase^66^. β-alanine is a molecular intermediary of GABA and glycine with a very similar mechanism of action, and is considered an inhibitor neurotransmitter^67^. In the brain, however, the best characterized pathway leading to β-alanine synthesis is the deamination and carboxylation of the pyrimidine uracil^68,69^, while very little is known about N-acetyl-β alanine dehydrogenase. Thus, the functional significance of the upregulation of N-acetyl-beta-alanine during wakefulness remains unclear. Similarly, very little is known about changes in the levels of N-acetylglutamine, the acetylated analogue of glutamine, one of the main amino acids used in protein synthesis and an essential constituent of the diet.

To our knowledge, these data provide the initial measures of the metabolome in the extracellular space of B6 mice across the sleep/wake cycle and after sleep deprivation. The finding that many of these molecules vary significantly as a function of sleep and wakefulness is consistent with the interpretation that these molecules are lower-level phenotypes contributing to the regulation of EEG and behavioral levels of arousal. Another not mutually exclusive interpretation is that at least some of the state-specific changes in brain metabolome are correlates of changes in behavioral states. Causal experiments will be needed to distinguish between these possibilities. Untargeted and targeted metabolomic approaches are being used to help clarify anesthesia-induced alterations in brain chemistry^21^. Acknowledged challenges for metabolomic approaches include analyses and interpretation of large and complex datasets, exemplified in the present study by the measurement of 33 analytes common to both mPFC and M1 brain regions. Analytic issues include the fact that metabolomic data violate the assumption of independence assumed by the general linear model. Finally, the present data are limited to the cerebral cortex. As metabolomic approaches become more widely used, it will be possible to build conceptual models that incorporate data regarding chemical networks across brain regions. These and other limitations are offset by the fact that metabolomic approaches, even in this early stage of application to studies of sleep, offer more complete insights than do measures of a single analyte. Medications used to modulate levels of arousal alter the entire chemical milieu of the central nervous system, not merely a single neurotransmitter. Future use of metabolomic approaches coupled to machine learning offers exciting opportunities to advance understanding of state-specific brain chemistry.

## Materials and Methods

### Animals

Adult, male C57BL/6 J (B6, wild type) mice (25 to 30 g; n = 19) were used. All animal procedures and experimental protocols followed the National Institutes of Health Guide for the Care and Use of Laboratory Animals^70^ and were approved by the University of Wisconsin-Madison licensing committee. Animal facilities were reviewed and approved by the institutional animal care and use committee (IACUC) of the University of Wisconsin-Madison and were inspected and accredited by association for assessment and accreditation of laboratory animal care (AAALAC).

### Stereotaxic Surgery

Implantation of recording electrodes and microdialysis guide tubes was performed under isoflurane anesthesia (2% for induction, 1 to 1.5% for maintenance). Each mouse was implanted with two microdialysis guide cannulae (Fig. 1a). One cannula was aimed for the left medial prefrontal cortex (mPFC) using stereotaxic coordinates Bregma AP + 1.94, ML − 0.3^71^. A second cannula was aimed for the right primary motor cortex (M1) with stereotaxic coordinates of AP + 1.5, ML + 2^72^. For electroencephalographic (EEG) recordings, screw electrodes were implanted over left and right parietal cortex (AP − 2, ML +/− 2) and cerebellum (reference electrode). To record the electromyogram (EMG), two stainless steel wires were implanted into the nuchal muscles. Cannulae and electrodes were fixed to the skull with dental cement. After surgery, mice were individually housed in transparent Plexiglas cages. Environmental lighting provided a 12 h:12 h light/dark cycle (lights on at 8 am; 23 ± 1 °C) with food and water available *ad libitum* and replaced daily at 8 am. Approximately one week was allowed for recovery from surgery, and microdialysis experiments started only after the temporal organization of sleep and wakefulness had normalized.

### EEG and EMG Recordings for Quantifying States of Sleep and Wakefulness

For one week after surgery the mice were conditioned for sleep studies by connecting them via a flexible cable to a multichannel neurophysiology recording and stimulation system that enabled EEG and EMG recordings (Fig. 1b) (Tucker-Davis Technologies Inc.). EEG and EMG signals were amplified and filtered as follows: EEG, high-pass filter at 0.1 Hz; low-pass filter at 100 Hz; EMG, high-pass filter at 10 Hz; low-pass filter at 100 Hz). All signals were sampled and stored at 256 Hz. States of sleep and wakefulness were scored off-line in 4-sec epochs using standard criteria.

### Microdialysis Experiments

The day before the experiment, each mouse was briefly anesthetized with isoflurane and the microdialysis probes (15 KDa pore size) were inserted into the guide cannulae^23^. Microdialysis probes in the mouse homologue of mPFC and M1 were connected to the microfluidic circuit (Fig. 1b) and perfused with artificial extracellular fluid (aECF) comprised of mM concentrations of: NaCl 147, KCl 2.7, CaCl_2_ 1.2, MgCl_2_ 0.85. The microperfusion pump (CMA/400, CMA Microdialysis), maintained aECF flow rate of 1.1 µL/min. Experiments were conducted from 7 am until 6 pm or from 7 pm until 6 am, during which time microdialysis samples from each cortical probe were collected every 15 min in 250 μL plastic vials (ESA, PN 70–1695; ESA, Inc.). At the conclusion of each dialysis experiment, microdialysis sites were histologically confirmed to be located in the targeted brain area. Dialysis samples were stored at −80 °C until they were shipped to the University of Tennessee to be analyzed using Ultra Performance Liquid Chromatography-High Resolution Mass Spectrometry (UPLC-HRMS) and an established metabolomics method^73^. At the University of Tennessee samples were also maintained at −80 °C until they were thawed and measured.

### Experimental Groups

Three different experimental groups were used (Fig. 1c). Mice are polyphasic, and thus can spend a substantial amount of time awake during their major sleep phase (day) and asleep at night, during their major wake phase. To standardize and consolidate sleep and wake episodes mice were first placed on a slowly rotating treadmill that kept them awake for 2 h. Dialysis samples collected during the second hour served as baseline measurements for standardizing the samples collected during the following 9 h of the light period (first two groups) or of the dark period (third group). Figure 1d shows the percent of time mice in each of the three groups spent awake during each 15-min sampling bin.

#### Group 1: Sleep for 6 h followed by enforced wakefulness on treadmill for 3 h (S6-EW3; n = 7 mice)

At 7 am, one hour before lights-on, each mouse was placed on a slowly moving treadmill (speed ≈ 0.7 cm/sec) and the flow for microdialysis probe was activated (1.1 µL/min). The mouse was awake while slowly moving on the platform, and five microdialysis samples were collected during the second hour to serve as reference. The mouse was then returned to its cage and allowed to sleep for 6 h. After ad libitum sleep, at 3 pm the mouse was placed back on the slowly moving treadmill for 3 h, to obtain EEG recordings and microdialysis samples during a “standardized” period of wakefulness, in which the mouse behavior was as homogenous as possible.

#### Group 2: Enforced wake for 6 h followed by sleep for 3 h (EW6-S3; n = 6 mice)

As described above, mice were placed on the slowly moving treadmill starting at 7 am and the second hour was used as reference. Mice were then returned to their cage and kept awake by exposure to novel objects for 6 h, followed at 3 pm by ad libitum sleep for 3 h.

#### Group 3: Spontaneous wakefulness for 6 h followed by enforced wakefulness on treadmill for 3 h (SW6-EW3; n = 6 mice)

Mice were placed on the slowly moving treadmill for 2 h starting at 7 pm, one hour before lights-off, and the second hour served as reference. At 9 pm mice returned to their cage and as expected, were spontaneously awake for most of the time. A novel object was placed in the cage every ~30 min to help maintain a sustained period of wake, but if the mouse slept, it was allowed to do so and microdialysis samples were not used for subsequent analyses. At 3 am mice were placed back on the treadmill for 3 h.

### Untargeted metabolomics using UPLC-HRMS

The present study used an untargeted approach to profile the metabolome of each dialysis sample. This technique provided information both for metabolites of known structure, which were identified as outlined below, and for metabolites with undetermined structure. Prior to untargeted analyses, a 15-μL aliquot of each microdialysis sample was diluted to 50 μL of total volume using Ringer’s solution. Instrumentation for untargeted metabolomics analysis included an Ultimate 3000 LC pump in line with an Exactive Plus Orbitrap™ mass spectrometer (Thermo Scientific). Ultra-performance liquid chromatography (UPLC) was accomplished under previously established chromatography^74^ using a Synergi Hydro-RP column (100 mm × 2.1 mm, 2.5 μm, 100 Å). Metabolites were detected using high-resolution mass spectrometry (HRMS) in negative mode with an electrospray ionization (ESI) source.

Following detection, raw data were compiled using the Metabolomic Analysis and Visualization Engine (MAVEN) software package^75^. Known metabolites were selected from a pre-existing list of ~300 compounds based on exact mass and previously determined retention times^73^. Metabolite selection resulted in the reliable identification of more than 40 small molecules in more than 80% of subjects; Group 1 (31 ± 16, mean ± SD), Group 2 (47 ± 8), and Group 3 (45 ± 5). Additionally, MAVEN enabled the extraction of all spectral features detected during analysis. Each spectral feature refers to a potential metabolite with an exact parent mass detected at a specific retention time. After removal of “known” metabolites and carbon-13 isomers, the remaining spectral features were classified as “unknown”. Total unknown features: Group 1 (2436 ± 850, mean ± SD; range, 998–3755), Group 2 (2388 ± 1529; range, 388–5016), and Group 3 (2297 ± 846; range, 947–2899).

### Classification and Statistical Analysis

#### Pre-processing

To promote generalizability of the results, the analysis was restricted to metabolites that were observed at least in 3 mice per treatment condition. This selection criterion was applied separately to each brain region and provided a list of 33 known metabolites common to both regions (mPFC and M1), and 3 metabolites that were unique to mPFC (36 metabolites total; Fig. 2). Raw ion intensities were standardized into z-scores using the means and standard deviations estimated from the baseline samples; note that baseline corrections were performed separately for each mouse, brain region and metabolite combination. Next, outliers were detected and removed from the data set; an observation was classified as an outlier if its z-score was more than 5 times greater than all other observations for the mouse/brain region/metabolite. In total, 13 out of 34302 observations (less than 0.04%) were classified as outliers. The observations were then coarse-grained to a time-scale of 1 hour (e.g., averaging 4 consecutive values from the 15-min samples), reducing the data set to 9 time-points. This served to reduce the variability in the data, as well as the number of missing data points. Missing data occurred when a metabolite was not detected in a microdialysis sample, when data were omitted due to a mouse being in the “wrong” behavioral state (for example, for a mouse in Group 1, a 15-min sample during the first 6 hours was discarded if during that time interval the mouse had been more than 50% of time awake rather than asleep), or when an observation was removed as an outlier. After coarse-graining, the remaining missing data were imputed in the following manner: 1) for metabolites that were completely absent from a sample, missing values were estimated by the mean z-score for that metabolite/time point across all other mice in the same experimental condition; 2) the remaining missing data points were estimated using the k = 2 nearest neighbors algorithm and averaging the observations for the two nearest time-points within the corresponding mouse/metabolite/brain region.

In total 1960 missing observations were interpolated to complete the data set (199 completely missing metabolites with 9 time-points each; 156 individual missing observations and 13 outliers), making up 16.6% of the total data set. It is worth noting that three mice (all in the S6-EW3 condition) contributed 66% of the missing values (1299 out of 1960). On further inspection, no technical reason why the samples from these three mice should be excluded was found. Furthermore, removing these mice from the analysis made no qualitative differences to the results. We thus conclude that the bias for missing data to be in the S6-EW3 condition does not adversely affect the outcomes of the analysis, and all results are reported from the fully imputed data set.

The unknown spectral features were clustered to determine whether the same peak was identified across multiple samples. Two observations were considered to measure the same unknown feature if their masses differed by less than 0.0014 and the retention times differed by less than 0.3. After clustering the features, we then applied the same preprocessing pipeline as described above for the known metabolites, resulting in a list of 39 unknown features (33 common to both mPFC and M1, and 3 unique features in each region).

#### Linear Mixed Effects Model

In order to determine whether there were statistically significant effects of prior treatment conditions and time on levels of measured analytes data were analyzed using LME models^76,77^. LME models are one of the few statistical techniques that are appropriate for analyzing repeated measures data, as most traditional parametric statistical techniques rely on the assumption of independence between data points. The other common approach for this type of data is a repeated measures ANOVA, but the LME analysis is more flexible with respect to missing data, and our analysis included many missing values, due to the absence of a compound at a specific time point or brain region, and/or due to a mouse that did not meet the criteria for sleep or wakefulness at a given time point.

A linear mixed effect model includes both “fixed” and “random” effects, allowing to determine the effect of a variable with a fixed number of states (in our case, the 3 behavioral conditions of either sleep, spontaneous wakefulness or enforced wakefulness), while simultaneously controlling for the effects of another variable for which only a random sample of possible values are observed (e.g., a random sample of mice). A mixed effect model has the form

$${{Y}}_{{i},{t}}={{\beta }}_{0}+{{b}}_{{i},0}+\sum _{{p}=1}^{{P}}{{\beta }}_{{p}}{{X}}_{{i},{t},{p}}+\,\sum _{{q}=1}^{{Q}}{{b}}_{{i},{q}}{{Z}}_{{i},{t},{q}}+{\epsilon }_{{i},{t}}$$

where,

$${{b}}_{{i},{q}}\sim {N}(0,\,{{\sigma }}_{{q}}^{2}),$$

and

$${\epsilon }_{{i},{t}}\sim {N}(0,\,{{\sigma }}^{2}).$$

In this model, Y_i,t_ is the response variable, in our case the compound sample of the i^th^ mouse at the t^th^ time point. The residuals ϵ_i,t_ were assumed to be independent and normally distributed with constant variance. To ensure the assumptions of normality and constant variance were satisfied, response values for some compounds were transformed using a log or square root transformation. The β_p_ values correspond to the fixed effects in the model and b_i,q_ are the random effects, which are assumed to be normally distributed, with mean zero and constant variance. The design matrices X and Z contain the values for each fixed and random explanatory variable respectively. Parameters for the LME models were estimated numerically using the lme4 library for the R software package^78^.

A model selection procedure was applied separately for each metabolite. Following^79^, we included as many random effects terms in each model as the data supported; when the variance of a random effect went to zero or two random effects became perfectly correlated then the model was over parameterized. Once the structure of the random effects was determined, the Akaike Information Criterion^80^ was used to select the set of fixed effects in the model.

Statistical analysis of the LMEs was performed using likelihood ratio tests, comparing the full model as identified by the model selection procedure with a reduced model with the effect of condition removed. Depending on the specific form of the fixed effects in the model, this could result in a test for a main effect of condition or a condition x time interaction.

An additional LME model was fit for each compound to study the concentration of metabolites at different time windows during the sleep experiments. Specifically, we defined a ‘sleep condition’ as a categorical fixed effect with 3 levels: “early” (first 3 h of the S6 experiment), “late” (last 3 h of the S6 experiment), and “recovery” (3 h of sleep after the EW6 experiment). The LME models were fit with sleep condition as a categorical fixed effect, time as a linear fixed effect, and mouse as a random effect. A likelihood ratio test was used to test for a significant interaction between sleep condition and time, that is, whether the trend for metabolite concentration (increasing, decreasing, constant) was different during the different sleep conditions.

#### Classification analyses

Multiple classification models were employed to determine whether microdialysis measurements could be used to accurately classify behavioral state sampling epochs as sleep, spontaneous wakefulness or enforced wakefulness. These analyses used the combined data from the mPFC and M1 brain regions to classify the metabolic profiles of sleep (S6), spontaneous wakefulness (SW6) and enforced wakefulness (EW6). One classification model employed was a partial least squares discriminant analysis (PLSDA), computed using the DiscriMiner package (https://www.rdocumentation.org/packages/DiscriMiner) in R, with figures generated using the ggbiplot package (https://www.rdocumentation.org/packages/ggbiplot).

A unique function of this technique is the feature classification model that produces a list of variable importance of projection (VIP) scores for each metabolite. As a sparse classification model, we considered logistic regression with an ℓ2 regularization penalty, implemented using the Python package scikit-learn. In problems with many more variables than observations, a sparse model prevents overfitting and improves prediction accuracy.

Prediction accuracy was assessed using a repeated 3-fold stratified cross-validation. Briefly, the population was divided into 3 groups, with the number of mice from each condition approximately equal for each group. Two of the groups were used to train the models (training set) and the remaining group was used to test the accuracy (test set). The process of dividing the mice into groups was repeated 100 times, and the prediction accuracy was estimated as the average across all repetitions. Significance testing for the classifiers was performed using a nonparametric permutation test. The condition labels for the mice were randomly permutated to remove any distinctions between the groups, and then the classifiers were reapplied, capturing the expected accuracy under the hypothesis of no differences. This method captures both the fact that chance levels of accuracy are 50% (33% for 3-way classification) and the structure of the design matrix, i.e., the number of predictor variables compared to the number of observations. The structure of the design matrix is important to consider because with so many predictors, one might expect to do better than chance even in the absence of a true difference between groups.

### Data availability

All relevant data are available from the authors upon request.
